# Influence of Smart Sensors on Structural Health Monitoring Systems and Future Asset Management Practices

**DOI:** 10.3390/s23198279

**Published:** 2023-10-06

**Authors:** D. M. G. Preethichandra, T. G. Suntharavadivel, Pushpitha Kalutara, Lasitha Piyathilaka, Umer Izhar

**Affiliations:** 1 School of Engineering and Technology, Central Queensland University, Rockhampton, QLD 4702, Australia; t.suntharavadivel@cqu.edu.au (T.G.S.); p.kalutara@cqu.edu.au (P.K.); l.piyathilaka@cqu.edu.au (L.P.); 2 School of Science, Technology and Engineering, Moreton Bay Campus, University of the Sunshine Coast, Moreton Parade, Petrie, QLD 4502, Australia; uizhar@usc.edu.au

**Keywords:** smart sensors, sensor networks, asset management, Structural Health Monitoring, SHM, artificial intelligence, machine learning, digital twin

## Abstract

Recent developments in networked and smart sensors have significantly changed the way Structural Health Monitoring (SHM) and asset management are being carried out. Since the sensor networks continuously provide real-time data from the structure being monitored, they constitute a more realistic image of the actual status of the structure where the maintenance or repair work can be scheduled based on real requirements. This review is aimed at providing a wealth of knowledge from the working principles of sensors commonly used in SHM, to artificial-intelligence-based digital twin systems used in SHM and proposes a new asset management framework. The way this paper is structured suits researchers and practicing experts both in the fields of sensors as well as in asset management equally.

## 1. Introduction

Structural Health Monitoring (SHM) has become an important part of many engineering fields. It is desirable to detect the defects in the structural members at an early stage so that possible remedies can be applied with significantly less cost. Early detection of the defect will also help to reduce the disturbance for the usage of the structure while repairing is undertaken.

SHM of assets including civil and mechanical structures on land, road and rail infrastructures, aerospace structures, naval structures, offshore fixed structures, etc. has become more convenient with the development of low-cost and easy to network sensors [1,2,3,4]. SHM helps in fine-tuning the scheduled maintenance frequencies as well as carrying out required repair or maintenance work before the schedule if there is a deteriorating condition in a structural element. This kind of data-driven smart SHM systems are cost-effective in the long run even though they increase the initial cost of the infrastructure. This also improves the safety of the structure for the users, reducing the probability of catastrophic failures.

The data-driven SHM systems provide a wealth of real-time information which will support the asset management system to make decisions based on the actual condition of asset elements. Wireless sensor networks play a significant role in collecting data from smart sensors in SHM systems and continue to grow [2,5]. The Internet of Things (IoT) and cloud-based systems have contributed a lot in developing modern smart SHM systems [6,7]. The conventional sensors do not have any intelligence integrated into them. They have only the sensing element that can convert a variation in physical parameter into an electrical signal; therefore, they just provide the measurement data. In contrast, the smart sensors essentially have the sensing element, a signal conditioning circuit, a microcontroller, and a communication circuit [8]. They are sometimes capable of self-checking for sensor performance and reporting if the sensing element is out of calibration. Furthermore, they do not need to transmit all the raw data, instead they preprocess the measurement data and reduce noise and unreliable datapoints from the transmitted data stream, which significantly reduces the data transmission between the sensors and base station.

For SHM, both destructive and non-destructive methods are used. Destructive Structural Health Monitoring involves methods that physically damage or alter a structure to obtain precise data about their condition, often employed when structures are no longer in service. It includes tests that detect structural failures and sample removal, offering high-precision testing data but at a high cost and with significant disruption.

Non-Destructive Structural Health Monitoring uses remote sensing and continuous monitoring techniques to assess structures without causing any harm. These techniques are typically employed for in-service structures to monitor their health over time without causing any damage. Non-destructive methods often rely on sensors, cameras, or other remote monitoring devices to assess the structural condition without physically touching or altering the structure. They also enable continuous or periodic monitoring, providing data over extended periods to track changes in the structure’s condition.

Recent trends in SHM lean more towards non-destructive methods due to advancements in sensors and data processing technologies [1,9,10,11]. Advances in sensor technology and data analytics have made it easier to collect and process large volumes of data from non-destructive monitoring. This data can be used to gain insights into structural performance and make informed decisions about maintenance and repairs.

Asset management systems have greatly benefited from recent advancements in non-destructive Structural Health Monitoring (SHM) practices. By analyzing data from non-destructive sensors, asset managers can implement predictive maintenance strategies. Predictive maintenance utilizes data to identify patterns and predict when assets may require maintenance or repairs, reducing downtime and maintenance costs. Non-destructive sensors can serve as early warning systems by detecting anomalies or deviations from normal operating conditions. This allows for proactive responses to prevent asset failures or accidents. For these reasons, this review paper focuses exclusively on smart sensors that can be utilized for non-destructive structural health monitoring.

The primary contribution of this paper is to introduce smart sensor technologies used for non-destructive SHM and propose an intelligent data-driven asset management framework using the data produced by non-destructive sensors. The paper is structured as follows: it begins with introductions to various non-destructive sensors and their operational principles in Section 2. Section 3 focuses on the integration of these sensors to realize automated structural health monitoring. Use of artificial intelligence to analyze data gathered from such automated sensing systems and maintenance prediction is discussed in Section 4. Lastly, in Section 5 an asset management framework is proposed to develop a decision-support system for businesses in assessing maintenance schedule and maintenance cost for structural assets.

## 2. Different Sensor Technologies

Traditionally, the SHM was undertaken by visual inspection, which often depends on the experience and skills of the inspectors and has possibilities for some defects going undetected at the early stage.

The new technologies used in SHM can effectively address the following challenges:

- Acquiring more reliable data;
- Accessing the locations remotely;
- Less disturbance for the usage of the structure during the inspection;
- Ability to detect both internal and surface defects;
- Digital transmission and storage;
- Data can be effectively used to predict the severity of the defect and the remaining life time of the member.

To enable these, the modern SHM systems employ different sensors based on different physical phenomena. They include ultrasound sensors, mechanical sensors, laser sensors, optical sensors, ground-penetrating radar sensors, electrical sensors, and micro electromechanical sensors. These sensors are discussed in the following sections.

### 2.1. Ultrasound Sensors

**Figure 1:**
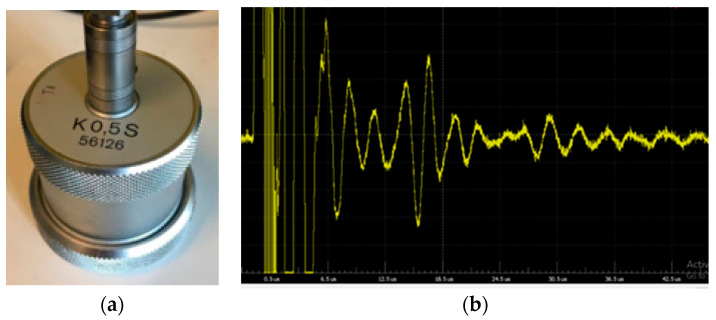
(**a**) An ultrasound sensor; (**b**) A-Scan from an ultrasound sensor.

Ultrasound sensors, also known as ultrasonic sensors, are commonly used in concrete assessment, and testing due to their ability to provide valuable information about the internal structure and properties of critical concrete infrastructure. Ultrasound sensors work by emitting high-frequency sound waves (typically above the range of human hearing, around 20 kHz to several MHz) using a transducer (see Figure 1a) into the material that needs to be tested. The transducer can be piezoelectric, magnetostrictive, or based on other technologies. When the ultrasonic waves encounter the concrete material, they propagate through it. The speed of these waves is determined by the material’s elastic properties, including its density, stiffness, and porosity. In concrete, these properties are closely related to factors such as compressive strength, density, and moisture content. As the ultrasonic waves encounter interfaces between different materials within the concrete such as aggregates, air voids, cracks, and internal defects, part of the energy is reflected back to the sensor/receiver module, while the rest of the energy is transmitted deeper into the material. The time it takes for the waves to return provides information about the distance to these interfaces. The returning echoes are detected by the sensor/receiver module and converted into electrical signals. These signals are referred to as A-scans and a typical A-scan is shown in Figure 1b. Multiple A-scans are then combined to form B-scans by moving the sensor, resulting in a cross-sectional scan of the testing area. C-scans provide a 3D view of the test area, which is constructed by combining several B-scans. By analyzing the time delay between the transmitted and received signals, as well as the amplitude and frequency characteristics of the echoes, it is possible to determine the depth and nature of internal features like cracks, delaminations, and voids. The sensor can also provide information about the concrete’s overall quality and homogeneity. The sensor’s output data is typically processed and analyzed by specialized software or equipment. These data can be presented in the form of visual representations, such as B-scan or C-scan images, which help visualize the internal structure of the concrete. Experienced technicians and engineers interpret these images to identify potential defects or irregularities.

Ultrasound-based non-destructive evaluation methods also have some limitations. The accuracy of measurements can be affected by factors such as surface conditions, temperature, moisture content, and the presence of coarse aggregates. Moreover, achieving proper contact between the sensor and rough surfaces can be challenging, potentially weakening the strength of the reflected echo signal. The interpretation of results requires expertise to differentiate between different types of internal features.

#### Application of Ultrasonic Pulse Velocity

Ultrasonic Pulse Velocity (UPV) is one of the widely used non-destructive condition assessment methods used in concrete structures. This method is commonly used to evaluate the consistency or homogeneity of the member and detect voids or cracks or internal defects in the member. However, it can also be used to determine the crack depth and condition monitoring of a member over time. In addition, the UPV measurements can be used to determine the porosity of mortar in the laboratory condition [12].

In common application of an ultrasonic pulse, a p-wave (compression wave) is used to measure its velocity through the material. Based on the IS13311-1:1992 the quality of the concrete is classified as shown in Table 1.

IS13311-1:1992 [13] also proposed an equation to determine the dynamic modulus of elasticity of the concrete using the pulse velocity measurements. Considering the accessibility and mobility of the equipment this test is considered one of the effective techniques for evaluating the quality and internal defects of the concrete. However, several field conditions such as surface conditions, moisture condition of the concrete, stress level in the concrete members and reinforcement bars may influence the measurement of the UPV.

The UPV will travel at 1.2 to 1.9 times faster in steel compared to concrete; therefore, often, the first waves reaching the receivers are partially travelled through the steel. The addition of silica powder to concrete is used to enhance the strength of the concrete. Due to the fine particle size, the silica fills very small voids in the concrete and also increases the UPV reading [14]. This increase is a direct reflection of the improved quality of the concrete rather than any influence of silica on the pulse velocity.

On the other hand, plastic will absorb the pulse waves, which can result in lower UPV readings in a concrete that contains large amounts of plastic-related aggregates such as PVC fibers and recycled plastics. In concretes with PVC fibers, the pulse velocity starts to decrease when the PVC fiber content is more than 0.5% [14]. Similarly, a higher volume of fly ash also adversely impacts the UPV measurement but an increase in curing time is found to reduce that effect [15].

While the non-traditional aggregates and reinforcements influence the UPV measurements, it is still an effective technique for continuing structural health monitoring of concrete structures, which often requires any changes in the reading. In addition, the calibration of UPV measurements against non-traditional admixtures through further research will standardize the UPV application across these concrete quality checks.

Janku et al. [16] compared the three different NDT for detecting delaminations in concrete bridges and found that the UPV misses the smaller targets (shallow cavities) or overestimated them compared to the infrared thermography methods.

Although there are many different techniques that are used and accepted in structural health monitoring, the pulsed system has been found to be one of the most effective and widely accepted equipment in the SHM [17].

### 2.2. Mechanical Sensors

**Figure 2:**
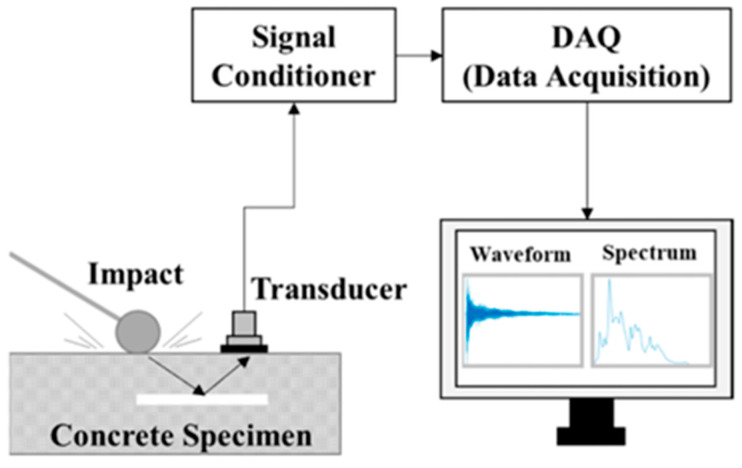
Schematic of Impact-Echo method [18].

The Impact-Echo method is another non-destructive testing technique that utilizes stress waves to assess the integrity and thickness of concrete structures. It is often used to detect flaws such as voids, delaminations, and cracks within concrete elements like slabs, walls, and columns. The method relies on the generation and analysis of stress waves caused by impacting the surface of the concrete structure as shown in Figure 2. A small mechanical or acoustic impact is applied to the surface of the concrete structure using a handheld hammer-like device, known as an impactor. This impact generates stress waves that travel through the concrete. The stress waves travel through the concrete in various directions. As they encounter interfaces between different materials or structural anomalies (such as voids or cracks), a portion of the energy in the waves is reflected towards the surface. A piezoelectric transducer or sensor is placed on the surface of the concrete close to the impact point. This sensor is capable of both emitting and receiving stress waves. It detects the stress waves that return to the surface after interacting with subsurface features. The reflected stress waves are recorded by the sensor and converted into digital electrical signals by using a conditioning circuit and a DAQ. The time between the impact and the reception of the reflected waves is measured. By analyzing the time of flight of these waves and their frequency content, the system can infer the depth and nature of anomalies or boundaries within the concrete. The data collected from the sensor is then processed and interpreted by specialized software. Analyzing the time and amplitude of the received signals helps in identifying the locations of subsurface flaws, such as delaminations, voids, and cracks.

Impact-Echo is advantageous because it is non-destructive and does not require direct access to the opposite side of the concrete. It can provide valuable insights into the internal conditions of concrete structures without causing damage. However, it is important to note that the accuracy and reliability of the technique depend on factors such as concrete composition, surface conditions, and the expertise of the operator.

### 2.3. Laser Sensors

**Figure 3:**
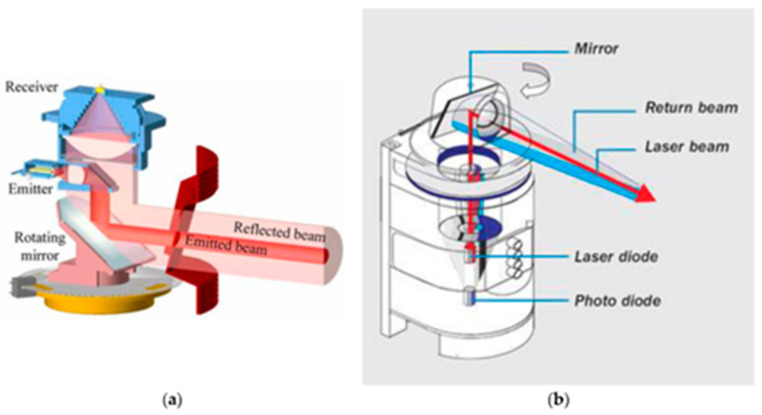
Typical 2D scanning mechanism used in (**a**) SICK 2D LiDAR, (**b**) SICK omnidirectional LiDAR [19].

LiDAR (Light Detection and Ranging) is a sensing technology that uses laser light to measure distances and create high-resolution, three-dimensional maps of objects and environments. LiDAR sensors work based on the principle of sending out laser pulses and measuring the time it takes for those pulses to return after bouncing off objects, as shown in Figure 3. These sensors emit rapid and short laser pulses in multiple directions using a rotating mirror, creating a laser beam that travels outward from the sensor. When the emitted laser pulses encounter objects, they are reflected toward the LiDAR sensor. The LiDAR sensor measures the time it takes for each laser pulse to travel to an object and back. Since the speed of light is constant, by knowing the time it took for the pulse to return, the LiDAR system can calculate the distance between the sensor and the object. LiDAR sensors emit laser pulses at a very high rate, often thousands to millions of pulses per second. This rapid emission and reception of laser pulses create a point cloud of distance measurements. The collected distance measurements are combined to form a point cloud. Each point in the cloud represents a specific location in space along with its corresponding distance from the LiDAR sensor. The point cloud data can be processed and analyzed to create detailed 3D maps of the environment. Advanced algorithms can be used to filter out noise, remove unwanted reflections, and generate accurate representations of objects and terrain.

LiDAR technology is used to detect deformations and cracks in structures. This technique does not need any contact with the structure; therefore, it is considered a faster and easily accessible technique used in SHM of larger structures and can also be used in structures with limited access. In addition, the measurement is not affected by the light sources; therefore, it can be effectively used in bridges, tunnels and roads/pavements. However, the use of LiDAR is affected by weather conditions and therefore, it is limited to surface defect detection only [9]. That means any non-structural components on the surface area need to be removed to obtain the correct accuracy results of LiDAR technology.

### 2.4. Optical Sensors

Fiber Optic Sensing (FOS) is an alternative technology to traditional SHM techniques. Over the traditional sensors, FOS has many advantages including low cost, less weight, low maintenance and is often used for continuous SHM [11]. They can be used in most of the infrastructures including offshore platforms as their measurements are not influenced by environmental conditions or vibrations. However, they application is mostly limited to detect local and subsurface defects unless it is embedded into the member. There are some experimental studies carried out on large structures for prolonged time to detect their structural changes over a long time [20].

**Figure 4:**
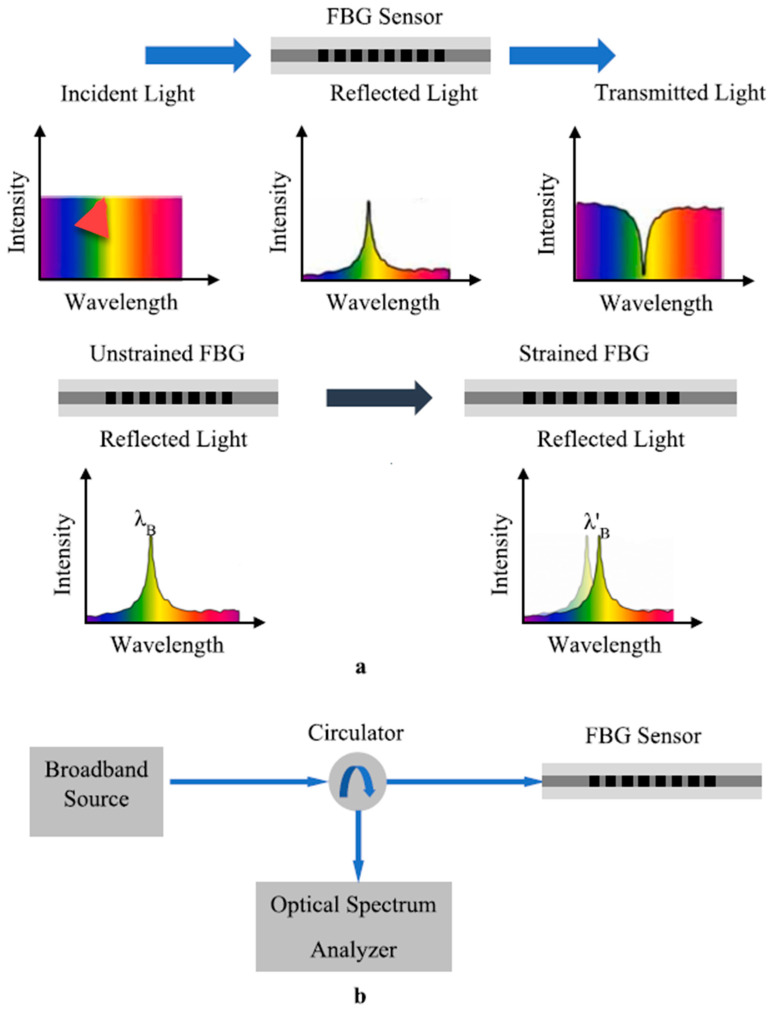
(**a**) Working principle of the FBG (**b**) Configuration of the FBG interrogation [10].

Most of the fiber optic sensors are based on the Fiber Bragg Grating (FBG) principle. Figure 4 illustrates the FBG sensor operating principle. A normal optical fiber has a core of material with uniform optical refractive index, where the FBG sensor is made by placing gratings at uniform intervals of Λ. If the refractive index of the optical fiber is $n_{2}$, the refractive index of the grating is $n_{2},$ and the effective refractive index of the FBG fiber sensor is $n_{e}$, then the Bragg wavelength ($\lambda_{B})$ can be written as $\lambda_{B}=2n_{e}\Lambda$. Therefore, the frequency of the reflected wave is fixed and dependent on the grating period Λ and $n_{e}$. However, when the optical fiber is subjected to a strain, the grating period changes and as a result the reflected wavelength will change. Figure 4a shows the transmitted spectrum and the reflected spectrum under normal conditions(upper three parts of the figure), and the lower two parts of Figure 4a show the frequency spectrum shift due to a strain in the FBG sensor. When the FBG sensor is attached to a structure, any strain in the structure can be measured using this spectral shift. Figure 4b shows the schematic diagram of the optical FBG instrumentation system.

### 2.5. Infrared Thermographic Sensors

**Figure 5:**
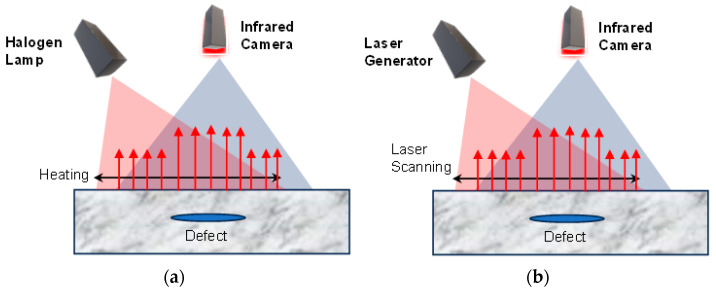
IR thermography using (**a**) Halogen lamp (**b**) Laser light.

InfraRed (IR) thermography is a simple, fast, non-destructive and practical technique for damage detection of structures on the surface or subsurface. As it is also a noncontact technique, it is very effective for composite materials to detect small defects on the surfaces and subsurfaces [11]. However, it cannot detect the depth of the cracks and is less effective in detecting internal defects. IR technique also cannot be used with highly reflective surfaces. Figure 5 shows IR thermography using two different energy sources.

He et al. [21] effectively used IR technology for vibration-based SHM. They reported an average error in natural frequency was less than 4% and the technique can be used in larger structures in harsh environmental conditions with limited access. Amjad et al. [22] evaluated the applicability of the IR in real-time SHM and the detection of fatigue cracks have reported that the IR technology can be used to detect the crack as small as 1 mm with loading frequency as low as 0.3 Hz. This shows that IR can be used in large-scale structures where the loading frequency is often less than 1 Hz.

### 2.6. Ground-Penetrating Radar Sensors

Ground-Penetrating Radar (GPR) is an imaging technology that uses electromagnetic waves (10–1000 Hz) to identify underground utility lines and reinforcement or tendon arrangements in the concrete members. This also can be used to identify the groundwater level and bedrock. The electromagnetic is reflected when they encounter any variation such as boundaries, different material interfaces and any infections in their paths. These reflected waves will be used to analyze to obtain the mapping of the subsurface profile. GPR has better penetrating capacity compared to UPV or IR technology; therefore, it can detect defects at larger depths [23].

**Figure 6:**
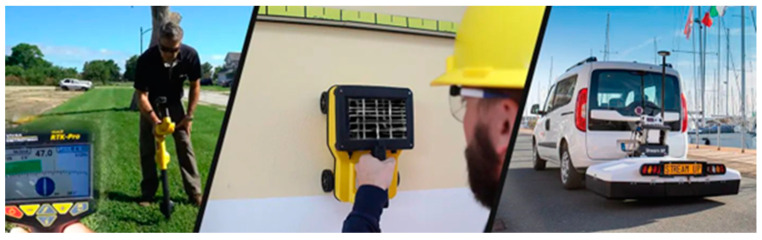
Use of Ground Penetrating radar (Picture curtesy of C R Kennedy Geospatial Solutions).

**Figure 7:**
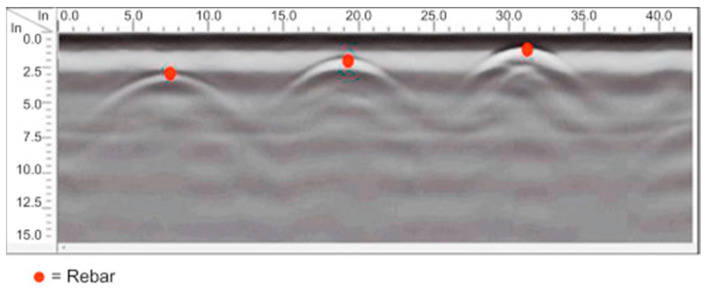
GPR used to locate reinforcement in the concrete [17].

In structural inspection, GPR is most commonly used for locating steel reinforcements/tendons, and mapping voids. With improved analytical capabilities the GPR is also effectively used for condition assessment of Fiber-Reinforced Polymer (FRP) applications in structures such as detecting delamination of FRP [17]. Figure 6 shows different uses of GPR sensors. Figure 7 shows the location finding of reinforcement in concrete using GPR.

The accuracy of the radar technology may be affected by overenthusiastic triggering, external noise, equipment failure, or malfunction. Different techniques such as trace editing (interpolation between good traces to compensate for any missed/bad data) or rubber-banding (modification of A-Scan traces) can be used to eliminate poor data collection [24].

The capability of GPR highly depends also on the effective mathematical model for predicting or detecting voids or different materials, which still requires more development to be used effectively in the SHM [25].

### 2.7. Electrical Parameter Measuring Sensors

**Figure 8:**
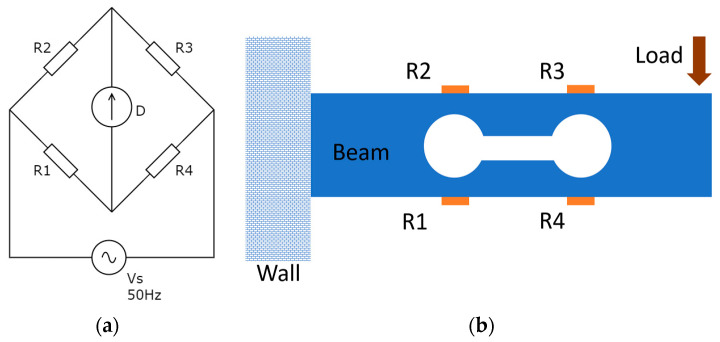
(**a**) The circuit diagram of the bridge. (**b**) Schematic diagram of the strain gauge load cell.

Electrical sensors are the most common type of sensors used in any smart sensor network as they are inherently easier to network and wirelessly connect with less additional hardware. The electrical parameter measuring sensors can be categorized into three types namely inductive, resistive, and capacitive (LRC) sensors. Resistive sensors are very common in the Wheatstone Bridge configuration to measure the deflection of structures. Figure 8 illustrates the circuit diagram and the schematic diagram of the strain gauge deflection measurement system. R_1_, R_2_, R_3_, and R_4_ are identical resistive strain gauges and D is the detector. The voltage across the detector D (V_G_) can be written as

(1) $$V_{G}=\left(\frac{R_{1}}{R_{1}+R_{4}}-\frac{R_{2}}{R_{2}+R_{3}}\;\right)$$

Under no load condition, all four strain gages have equal resistance and therefore, the voltage across D is 0 V. When the beam is loaded, the voltage output will be dependent on the variation in resistance of the strain gauges, where properly calibrated load cell output voltage can directly be converted in to strain value. There are more complicated resistive-type sensors developed by researchers for structural health monitoring [26,27,28].

Capacitive sensors are mostly constructed in parallel plate configuration or interdigitated (IDT) configuration. Piyathilaka et al. presented a capacitive-sensor-based 2D substrate imaging technology for the health monitoring of buildings [29]. In most of the IDT sensors the variation of capacitance is extremely low, so that it is necessary to have highly sensitive front-end electronics in the instrumentation system to measure these variations causing the SHM cost to be high. Preethichandra and Shida presented a method to measure very small capacitance changes in capacitive sensors with low-cost front-end electronics [30].

**Figure 9:**
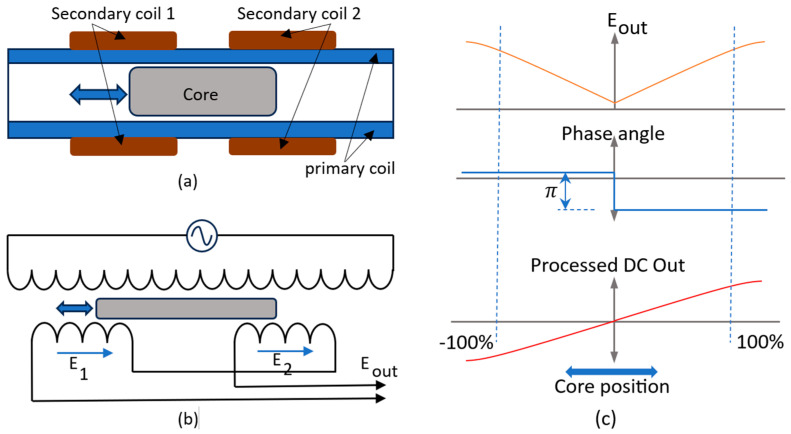
(**a**) Cross section of a LVDT (**b**) equivalent circuit diagram of the LVDT (**c**) signal output of the LVDT.

Inductive sensors are also commonly used in SHM systems. The Linear Variable Differential Transformer (LVDT) is one of the common types of inductive sensors used for displacement measurement. Figure 9a shows the cross-section of a LVDT and (b) shows the equivalent circuit diagram. In Figure 9c, two top sections show the magnitude and phase angle of the output voltage E_out_, while the bottom part shows the DC output voltage of the LVDT instrumentation system. The instrumentation system processes the magnitude and phase angle of the E_out_ signal in a way that the output DC voltage is a linear representation of the displacement from -100% to +100%. There are various advanced circuit designs for processing the LVDT signals to reduce the nonlinearity error [31,32].

Table 2 shows recently developed electrical sensors for structural health monitoring. The structural health monitoring of aerospace structures is a crucial issue in order to operate them safely. Qiu et al. proposed an impact monitoring system based on piezoelectric sensors (PZT) [33]. This is a continuous monitoring system with ultra-low power requirement and suitable for airplanes as well as spacecrafts. Yuan et al. presented a PZT-based guided-wave Gaussian process to evaluate the cracks in air plane structures [34]. There are number of microwave antenna-based surface crack detectors reported in the recent past [35,36,37]. They all measure the reflection coefficient (S_11_) in order to determine the cracks in the structure under monitoring. Ossa-Molina et al. reported a structural strain measurement system using S_11_ [38]. There are many different structural strain measurement techniques presented by other groups including measuring the voltage, capacitance, and frequency shift of injected signal [39,40,41]. Xie et al. presented a magnetostrictive patch guided-wave sensor for defect detection in metallic structures [42]. For all the above-mentioned electrical parameter measuring sensors, power from external source is necessary. However, in the recent past energy harvesting sensors for SHM becoming popular [43].

### 2.8. Micro-Electro-Mechanical Systems Sensors

Micro-electro-mechanical systems (MEMS) sensors and systems are functioning machines that are on a micro-meter scale. The functions of sensing, actuation, signal processing, and communication are integrated in MEMS sensors locally, thereby providing control of physical parameters at a micro level. These small sensors play an important role in structural health monitoring and are considered to be one of the main drivers in increasing the feasibility of implementing long-term smart (more than a few years) structural health monitoring solutions due to their small size, lower cost, and lower weight.

Sensors used for health monitoring can either be used at the surface of the structure or can be embedded inside the structure. Surface sensors (SS) are coupled to the outer surface of the structure. While easier to implement, SSs are exposed and can be susceptible to the surrounding environment. On the other hand, embedded sensors (ES) are installed or integrated in the structure during manufacturing and can help implement smart monitoring structures. MEMS sensors have the advantage that they can be implemented either as an SS or an ES sensor. The choice is usually application-dependent, but certain factors may need to be evaluated before choosing one type of sensing setup over the other. In structures, especially in composite materials where the presence of ES can modify the microstructure, SS can be a better option [45]. The packaging and service life of ES are important factors in working out the feasibility of such sensors [46].

Table 3 shows some of the areas where MEMS sensors are being used in structural health monitoring:

Crack detection can be carried out by different methods and sensing techniques including passive and active techniques. Passive techniques include measuring drift and listening to acoustic or ultrasonic waves using accelerometers and inclinometers. Active techniques introduce sensing of a guided microwave likely generated inside the structure by the sensing system. Flexible thin-film capacitive sensors have been used to detect and localize cracks in concrete in a laboratory setting [47]. Accelerometers and vibrometers are also used to monitor the structural health, where these sensors are used to either listen to acoustic emissions or ultrasonic waves. A MEMS vibrometer was proposed in [48] which was composed of a moving mass inside a reference frame. The sensor was aimed at detecting guided ultrasonic waves in lightweight concrete structures and tested on a custom-built test bed.

A MEMS sensor that simultaneously measures the temperature and moisture in concrete structures was reported in [49,50]. The sensor consisted of a microcantilever coated by temperature and moisture sensitive polymer. The sensor response was evaluated for short (24 h) and long term (230 days) after embedding it into the concrete. The sensor was protected by stainless steel jacket and polymer coating.

#### MEMS Sensor Design Scheme 

**Figure 10:**
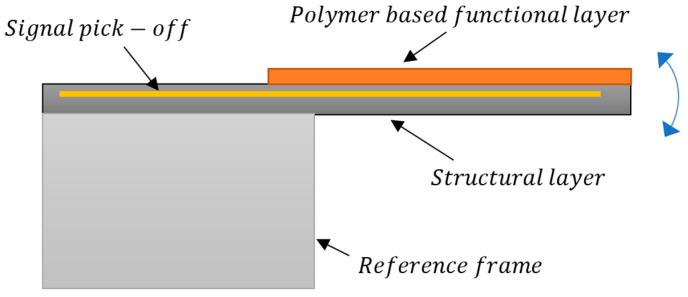
General schematic of a moving micro-cantilever to measure structural health parameters such as temperature and moisture.

**Figure 11:**
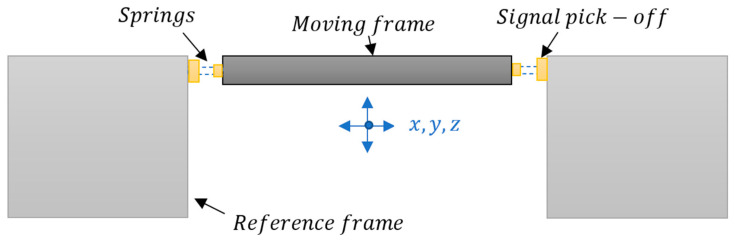
General schematic of a moving mass supported by springs to measure structural health parameters such as vibration, acceleration (drift), sound emissions due to crack formation.

As discussed above, many MEMS sensors for structural health monitoring are based on a moving mass which is fabricated either through bulk micromachining or surface micromachining. In bulk micromachining, the moving mass is released from the fixed substrate/structure using dry and wet etching techniques. The bulk of the substrate is etched from the backside to achieve the desired thickness of the mass. In surface micromachining the mass is achieved by depositing a layer of desired thickness over a sacrificial layer on the front side of the substrate which is then removed to create a moving mass. The moving mass is usually a micro-cantilever (see Figure 10) or mass suspended by springs (see Figure 11) with reference to a ‘fixed’ frame. These cantilevers are coated with relevant polymers which act as the sensing films (functional layers) that react to changes in target parameter. When the functional layer sees a change in temperature or moisture, it undergoes dimensional changes and tries to expand or contract. As the layer is physically restrained by the adjacent structural layer, they instead move upwards or downwards under strain. This multi-layer structure is usually embedded with a sensing layer that responds to the strain by a change in its resistance. This change can be calibrated to measure the target parameter, i.e., temperature, moisture, or vibrations. Due to the inherent capability of integrating different fabrication processes and materials in MEMS, functional layers sensitive to different environmental parameters can be combined to simultaneously measure multiple values. MEMS sensors based on moving cantilevers are reported for temperature and moisture sensing [50] and vibration sensing [51] in the literature. A typical setup for suspended mass sensors is mainly used in accelerometers, where a moving mass is supported to a reference frame by springs and the signal is picked off by capacitive fingers situated both on the reference frame and moving mass. Sensors which are based on moving cantilever masses are simpler to fabricate and are more sensitive than suspended-mass sensors but are limited to single axis sensing if used alone.

Another way of measuring change in moisture is through capacitive sensing, where a functional layer coated beam is sandwiched between two electrodes [52]. As moisture or humidity changes, water sensitive layer coated on a beam changes its dimensions thereby displacing the beam. This in turn changes the capacitance between the two electrodes.

## 3. Automated Systems for Structural Health Monitoring

In recent years, the integration of robotics in infrastructure monitoring has emerged as a transformative approach, revolutionizing the way we assess, manage, and maintain critical structures. The adoption of robotic systems for infrastructure monitoring has improved the efficiency, accuracy, and safety of the monitoring processes, enabling the timely detection of potential issues and facilitating proactive maintenance strategies [53,54,55,56]. This section discusses how different sensors are being used by robotic systems for infrastructure monitoring.

Vision sensors such as cameras and lidars have been used mainly with robotics drones, enabling the inspection of critical structures that were previously difficult to access through conventional means [9,56,57]. Drones have emerged as the preferred choice for deploying vision sensors due to their inherent advantages, such as compact size, maneuverability, and ease of hardware integration. Moreover, their agility allows them to navigate complex terrains and confined spaces, reaching areas that are otherwise inaccessible to human inspectors.

Primarily, drones are employed with cameras for aerial inspections, facilitating comprehensive surveys of large-scale structures such as bridges, towers, and wind turbines. Automatic detection of cracks and measuring them using machine learning and image processing is one of the highly researched areas [58,59,60]. In these methods, video footage is recorded and processed offline to automatically recognize cracks mainly in concrete structures The recent advancements in deep learning techniques improved the crack detection accuracies, but the onboard computing power is not sufficient to run these bulky models. One possible solution would be to stream the live videos to the ground station and process it there as proposed in [60].

LIDAR sensors have been used with drones to generate 3D models of critical infrastructure [9,56]. The LIDAR sensors mounted on drones emit laser pulses towards the ground or the infrastructure elements, and the time taken for the laser beam to bounce back to the sensor is measured. By collecting a multitude of such data points, LIDAR creates dense point clouds that represent the precise 3D shape of the infrastructure. These point clouds can be further processed using advanced algorithms to reconstruct the infrastructure’s surfaces, revealing fine details that might be otherwise challenging to perceive. LIDAR sensors and drones are used widely for building 3D models of bridges [61], tunnels [9] and in pipelines [62]. The 3D models generated through LIDAR-equipped drones provide a wealth of information for infrastructure assessment and maintenance. Structural engineers and experts can analyze these models to detect anomalies, measure dimensions, identify deformations, and assess overall structural health. These insights prove instrumental in making informed decisions regarding maintenance schedules, repairs, or even planning new infrastructure projects.

Thermal cameras have also been used in some applications with robotics aerial drones. One of the prominent use cases of thermal cameras on robotics drones is the inspection of solar farms [63,64]. Solar photovoltaic (PV) arrays are susceptible to various faults and malfunctions, such as hotspots, broken cells, or defective junction boxes. These faults generate heat variations that can be easily detected by thermal cameras.

Ultrasound-based infrastructure technologies have been effectively integrated with robotic technologies. However, the application of ultrasound techniques in robotic settings presents challenges, particularly in ensuring sound coupling between the sensor and the surface being assessed. In [65], researchers utilized ultrasound sensors to measure the thickness of a spray liner inside a pipeline. To maintain the coupling between the liner and the sensors, they applied a thin layer of water before taking the measurements. Subsequently, the data processing was conducted offline to determine the thickness accurately.

Ultrasound-based robotic technologies have found applications in many underwater scenarios, where the surrounding water serves as the medium for transmitting the ultrasound signal from the sensor to the surface. Specifically, ultrasound technologists, along with underwater drones, are primarily utilized to assess the condition of underwater pilings of bridges, underwater structures in harbors, and dams [55,66,67].

In recent years, Ground-Penetrating Radar (GPR) combined with robotics has emerged as a powerful and efficient approach for infrastructure monitoring. By integrating GPR with robotic platforms, infrastructure monitoring becomes more accessible, safer, and capable of delivering comprehensive assessments. Robotic platforms equipped with GPR technology has used for inspections of bridge structures, both above and below the surface. By traversing bridge decks and supporting structures, robots can assess concrete integrity, identify corrosion in reinforcement, and detect hidden defects, such as voids or delamination. The real-time data analysis capabilities of these systems allow for the immediate identification of critical issues, facilitating timely maintenance and minimizing potential risks.

Inspecting tunnels and underground structures can be challenging and hazardous for human inspectors. GPR-equipped robots have been proposed in previous research work as a safe and efficient solution for assessing tunnel linings, locating cavities or voids, and monitoring potential structural weaknesses [68,69,70]. The ability to access hard-to-reach areas and gather accurate data enhances overall safety and reliability.

The integration of robotic sensing for infrastructure monitoring presents several challenges that need to be addressed for successful implementation. Firstly, navigating complex environments with narrow spaces and obstacles can be difficult for robotic platforms, limiting their access to critical areas. Secondly, while sensors like Ground-Penetrating Radar (GPR) provide valuable data, they also have limitations in penetration depth and resolution, requiring careful data interpretation. Power management and endurance are other concerns, as continuous monitoring strains of robot batteries, necessitate frequent recharging and reducing coverage areas. Moreover, the vast amount of generated data requires efficient processing and real-time analysis capabilities, demanding powerful onboard computing or reliable communication links.

One of the major limitations of current drone technologies lies in their onboard data processing capabilities. Most drones lack the computing power required to conduct real-time data analysis during flight. Instead, the data collected by vision sensors, such as camera footage and LiDAR point clouds, are recorded and later processed offline. The recorded data is subject to post-flight analysis and algorithms specifically designed to identify abnormal conditions, such as cracks, bulging, and changes in colors or patterns that may signify structural damage or degradation. While this approach is effective in detecting anomalies after the flight, it does not afford real-time insights during the inspection process. As a result, time-sensitive situations, where immediate action may be required, may not receive prompt attention. However, ongoing advancements in robotics and artificial intelligence hold the promise of bridging this gap, aiming to equip drones with onboard data processing capabilities, enabling them to identify and respond to potential issues in real-time.

Ensuring robustness and reliability in harsh environments is crucial, as is establishing effective communication solutions for remote locations with limited network connectivity. Human–robot interaction interfaces must be intuitive and user-friendly for operators to supervise and intervene when necessary. Safety protocols are paramount to prevent accidents or damage to critical structures. Additionally, the initial setup costs and scaling for extensive monitoring require careful planning and budget considerations. Complying with regulations and obtaining necessary permits is vital to ensure legal compliance. Overcoming these challenges necessitates interdisciplinary collaboration and continuous research and development in robotics, sensor technologies, and artificial intelligence for successful and efficient robotic infrastructure monitoring systems.

## 4. Role of Artificial Intelligence in Structural Health Monitoring

Artificial intelligence (AI) and Machine Learning (ML) have become common things in almost every field due to the recent developments in computer hardware and data communication, where near-real-time processing of acquired data and fast retraining of neural networks became a reality [71,72]. Especially, edge AI plays a vital role in large-scale SHM and it has become a reality with current Internet of Things (IoT) devices where power and area efficiency have been optimized [73]. The use of AI and ML has increased the ability of detecting structural health issues of assets more precisely and in advance. The global demand for digital SHM systems is $2087.91 million in 2022 and predicted to be $6431.52 million in 2030 according to Vantage Market Research [74]. This shows the demand for intelligent SHM systems and most of them are now AI-based.

### 4.1. Artificial Intelligence and Machine Learning for Structural Health Monitoring

With the development of AI and ML focused hardware, SHM with nondestructive test (NDT) methods became a common practice [71,75]. Mondal and Chen had done a systematic review on optical camera-based, vibration-based and other NDT-based methods in SHM and predicted that NDT based unmanned aerial systems for SHM will have a compound annual growth rate of 57.5% from 2021 to 2028 [76]. There are many different techniques suggested for AI-based SHM, where ML-based systems have taken the lead in the recent years [77]. Decision tree (supervised), Support Vector Machine (supervised/unsupervised), k-Nearest neighbor (supervised), Bayesian (supervised), Neural Network (supervised/unsupervised), K-means (unsupervised), Gaussian mixture (unsupervised), and Association analysis (unsupervised) are few of the learning models commonly used in machine learning algorithms for SHM [78]. Luca et al. proposed a model order reduction and fully convolutional networks to analyze the vibration sensor data for a bridge SHM and reported that the accuracy is more than 85% [79]. Chen et al. presented an AI-based monitoring system for external disturbance detection and classification of a buried pipeline where they have used Quadratic-Support Vector Machine classifier with more than 99% accuracy and a Convolutional Neural Network(CNN) had been used for the testing phase and achieved an accuracy more than 96.1% [80]. These examples show the level of accuracy that can be achieved through AI- and ML based SHM.

All the above mentioned SHM systems are with fixed NDT sensors attached to the structures, which causes the cost of the system to be high. In contrast, Alzughaibi et al. proposed a community-based multi-sensory SHM system which comprises of smartphone camera and accelerometer data from occupants in the building [81]. The system was mainly setup to monitor the behavior of a building during earthquakes. They have shown through shake-table experiments that the developed system can achieve sub-millimeter accuracy. This is another new approach for SHM through community engagement.

### 4.2. Deep Learning for Structural Health Monitoring

Deep Learning(DL) is a subset of ML where there are more than three layers in the neural network(s) including the input layer and output layer [82]. The use of AI and supervised machine learning had been increased in the last decade or so, but with the availability of low-cost IoT enabled sensors, the number of such sensors placed in a SHM has increased by a large number producing a huge amount of real-time data. It became a challenge to train the networks with supervised learning algorithms, but at the same time developing unsupervised learning algorithms increased the demand for computation. The recent developments in CPU hardware were in favor of this and the demand for DL algorithms in SHM became high. The advancement in IoT enabled sensors and Edge-AI hardware were another boost to realization of DL-based SHM in the recent past [72,73]. Jayawickrama et al. presented a comprehensive review on optical fiber sensor based SHM systems with DL [10]. They have reviewed and compared a large number of recent different fiber optic sensor based SHM systems using DL algorithms. Azimi et al. have highlighted the importance of real-time data from the system for the DL to make more accurate decisions on damage detection in SHM [83].

There are recent developments of data-driven SHM systems using different deep learning models. For example, Dang et al. presented a feature fusion and hybrid DL system for SHM [84]. Seventekidis and Giagopoulos proposed a combined finite element and DL method [85]. They have a mixed bag of results from the experiments where multiple damages were identified with 100% accuracy, but small single damages were able to be identified with a minimum accuracy of 88% when the training validation accuracy was 90.76%. Kulkarni et al. proposed a DL augmented infrared(IR) thermography method for SHM of paved roads [86]. This method was tested by UAV-mounted IR camera images, and they have reported that their algorithm can detect damages at the interface of the road surface and road base, approximately 10 cm beneath the road surface. All the above examples of DL-based SHM systems have assumed that all the sensors employed are in good working condition. However, in reality there will be a few damaged or out of calibration sensors in a large system when the SHM systems get old. Hou et al. have proposed a unique method of DL and data augmentation based data imputation method for such situations [87].

### 4.3. Digital Twin in Structural Health Monitoring

**Figure 12:**
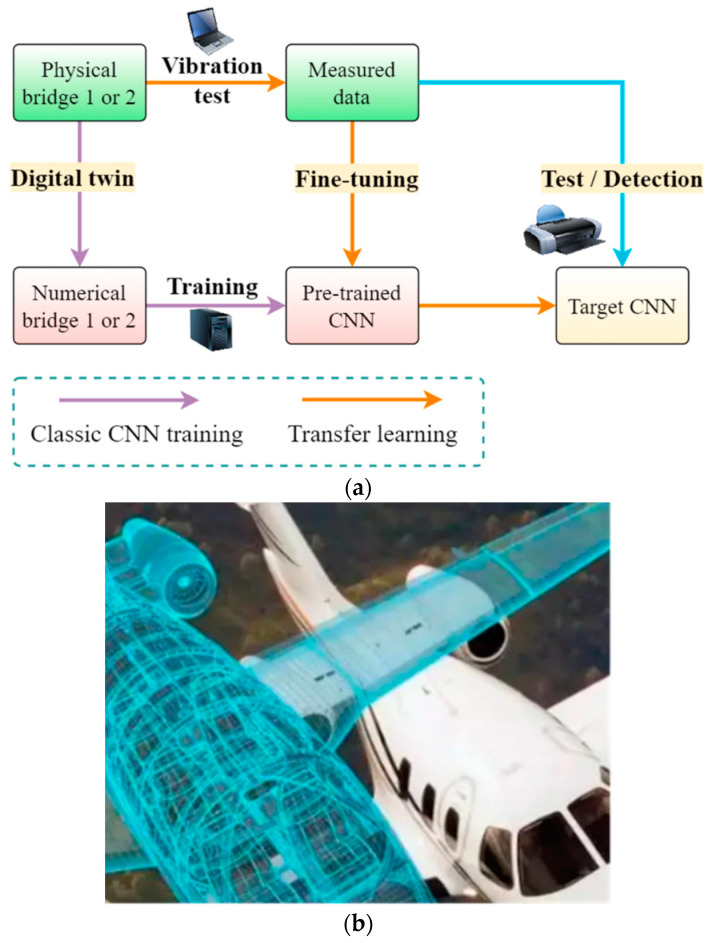
(**a**) Overview of DT technology for bridge condition monitoring [97] (**b**) Digital Twin of an aircraft structure for SHM [91].

Digital Twin (DT) is a hybrid of the traditional theoretical computer simulation system and a physical real-time monitoring system, where the actual data is fed to the digital simulation system to train it with more real-time information. DTs are becoming more common in modern civil infrastructure SHM systems as they provide more realistic predictions in real-time [88]. Figure 12 illustrates a typical DT system for bridge condition monitoring real-time data from the physical measurement system together with two CNNs working on the numerically simulated computer model in (a) and a DT model for an aircraft structural health monitoring system in (b). The initial training of the pre-trained CNN is done with a training dataset from the same bridge at the beginning under known loading conditions, and the real-time data is then fed to the pre-trained CNN. The outcome of the pre-trained CNN and the real-time data are then continuously fed into the target CNN for prediction of faults/deterioration of conditions.

The DT in SHM is not limited only to bridge structures, but the application of them can be found in smart cities and urban spaces, transportation including road, air and maritime transport, and energy systems including generators, windmills, and power transmission lines [89,90]. Furthermore, DT technology has been widely used by many major industries including aerospace industry [90,91]. Tuhaise et al. have identified data acquisition, data transmission, and digital modelling as the main technologies used in DT [92]; however, network security, data validation, neural network training with the latest data are also parts of the suit. Zhou et al. presented a non-neural network fuzzy-set-based joint distribution adaptation method for regression and online damage quantification for structural digital twin [93]. This method is very good if there are multiple structures of the same model, for example, a fleet of ships or aircrafts, where the base model DT can be used to monitor SHM of all individually without modifying it but, modifying the DT copy of asset under investigation with current data and compare with the original DT to schedule maintenance accordingly. Predictive maintenance against scheduled maintenance to reduce unnecessary maintenance work and carry out maintenance work before the schedule to avoid a catastrophic failure when there is a need are the main advantages of DT in structural health monitoring [90].

Current DT technology applications in maritime SHM have taken the field to a new level. The number of publications on DT in the maritime industry has shown and exponential increase by number of papers reaching 231 publications in 2022 [94]. Liu and Ren have demonstrated a rapid acquisition method for structural stress in SHM of a ship hull where they have achieved a maximum error of 0.0005% in structural yield strength [95]. Leng et al. have proposed a condition-based SHM system for off-shore wind jacket structures [96]. The submarine infrastructure of offshore wind turbines always faces harsh working environments due to unpredictable ocean conditions and this DT system will provide the opportunity to estimate the remaining lifetime of the asset more accurately.

Application of DT in aerospace industry has increased in recent years. In the past, having a ground physical twin system of a spacecraft was the standard, but with the successful development of digital twin systems the cost of launching, tracking and monitoring of a spacecraft has reduced by a significant factor [98,99]. Wang et al. presents a method for developing a 3D point cloud based digital twin system, which will bring down the cost further [100]. Lai et al. have developed a measurement-computation combined digital twin system for aircraft SHM [101]. They claimed that the proposed framework can combine the measured and computed data to build an accurate digital twin of the aircraft system. Another typical example is the DT-based aircraft landing gear management system was proposed by Zhao et al. and their system can reproduce the actual fault conditions occurred and fine-tune the pressure pump operating procedures under similar fault in the future for a safe landing [102].

Building Information Modelling (BIM) had been there for decades in the built environment and construction industry mainly used in the design and construction phases. Digital twin is widely becoming popular in the SHM of civil structures in the post construction stages. The DT is a promising system that can update the smart cities concept with real-time data processed using continuously trained DT framework to understand the changing ‘metabolism’ of the city in order to serve the resident and visitor needs in a better way [89,103]. Xu et al. proposed a digital twin system for building structural health monitoring by combining a BIM and a real-scene 3D model [104]. However, merging the BIM with real-scene 3D models faces the incompatibilities between different organizational protocols, geometries, and space definitions, which are to be resolved case by case. They have run a trial on the Old Hall of the Nanjing Museum as a case study and it has been successfully merged. Dang et al. have demonstrated a cloud-based DT for SHM using DL, where they have done case studies and shown that the accuracy of detection of bridge structural damages is more than 92% [105].

All these examples show us that the DT is an emerging tool in the SHM in multiple industries and has an influence on asset management of respective structures.

## 5. Paradigm Shift in Asset Management with Intelligent and Data-Driven Structural Health Monitoring Systems

According to Vanier and Rahman [106], Asset management is a business process and decision-support framework that (1) covers the extended service life of an asset, (2) draws from engineering as well as economics, and (3) considers a diverse range of assets. While the goal is established such, Neumann et al. [107] identify the core principles of asset management as follows:

- Asset management is policy driven.
- Asset management is performance-based.
- Asset management examines options and trade-offs at each level of decision-making.
- Asset management bases decisions on merit.
- Asset management maintains clear accountability.

Aligned with the goal and core principles, Too et al. [108] describes a generic asset management framework by three sequential processes including strategic analysis, strategic choice, and strategic implementation. Consequently, Federal Highway Administration (1999) [109] suggests seven major components for a generic asset management system as shown below:

- Goals and policies (reflects customer input);
- Asset inventory;
- Condition assessment and performance modeling;
- Alternatives evaluation and performance modeling;
- Short-term and long-term plans (project selection);
- Program implementation;
- Performance monitoring (feedback).

It is evident that the asset management is drawn from engineering as implied by the goal whereas it is performance based as implied by the core principles. Furthermore, strategic analysis through condition assessment and performance modelling leads to a generic asset management framework/system. Hence, asset and maintenance managers need to be aware of the condition monitoring techniques that are most appropriate to the assets under their care [110]. The current practice of building inspection is mostly governed by the terminology in building inspection defined by Construction Industry Council (1997) [111] which has drawn from the following areas: valuation, property purchase survey and valuation, building survey, elemental or specialist investigation, investigation prior to alteration, reinstatement cost assessment for insurance, stock condition survey, schedule of condition, schedule of dilapidations, measured survey, and inspection of buildings under construction. In contrast to a structural investigation, they interpret ‘Structural Survey’ as a survey which covers all visible and accessible parts of a building, including those which are not part of the structure such as the roof covering, windows, and drains.

Property surveys can be identified in two types: synchronic and diachronic [112]. Accordingly, synchronic survey is a snapshot assessment of a building and the way it all fits together at a particular moment in time. In contrast, a diachronic survey is a way of studying buildings in terms of how they change or evolve over time. The Institute of Public Works Engineering Australia (2006) [113] stresses on service organizations about the importance of having a clear knowledge of their assets and the performance of them. Lack of knowledge of asset condition will result in premature failure which can lead to serious consequences for organizations. According to (Institute of Public Works Engineering Australia 2006) [113], various condition monitoring systems can be applied to buildings:

- Visual assessments;
- Laser profiling/roughness meters;
- Life expectancy review;
- Manual inspections (operators);
- Protection (paint) thickness;
- Capacity modeling (for failure);
- X-ray;
- Concrete decomposition testing and core sampling;
- Power usage monitoring.

Aligned with the Construction Industry Council (1997) [111], visual assessments have been common in practice leading to a lack of opportunities to adopt advanced techniques such as structural health monitoring. The uncertainty-attributed data have encouraged using combined fuzzy logic and AI systems for asset management decision-making [114,115]. Routinely scheduled condition data play a major role in such decisions as they are utilized for planning and maintenance of repair of building components [116]. The service life of assets based on deterioration analysis is mainly relying with condition data and performed by two principal approaches in the current practice: deterministic and probabilistic [117].

In the context of bridges, Morcous et al. [118] found two unique models, stochastic and artificial intelligence (AI), which served for the probabilistic approach, while only deterministic models served for the deterministic approach. In another study, they state the suitability of those models, not only for bridges but also for infrastructure assets [118]. Dasu and Johnson differentiate these models by the driving force such that statistical and deterministic models are model-driven whereas AI models are data-driven [119].

Deterministic models are those for which condition is predicted as a precise value on the basis of mathematical functions of observed or measured deterioration [120]. Straight-line extrapolation, regression-based, or exponential deterioration model curves are often used for phenomena where relationships between components are certain [121]. Undoubtedly, deterministic models were the first in the application despite the limitations and reliability issues in deterioration prediction of infrastructure [122]. This is mainly due to simplicity in mathematical operations and direct relationship between the input factors and the output.

In contrast, probabilistic models are based on statistical theory and provide a more realistic approach to predict current and future conditions through a range of possible outcomes [120]. Among these models, Markov chain has been used for the analysis of various infrastructures such as bridges [123], waste water systems [124], stormwater pipes [125], and community buildings [126]. Positive signs of such models are due to the robustness to handle the output of ordinal data type and the probabilistic nature of the underlying deterioration process [122], whereas the models that are sensitive to noisy data [119,127] and the collected data are in subjective nature [122], adding to the list of negatives.

In comparison with deterministic and probabilistic models, AI models’ structure is determined by data, i.e., models are data-driven. AI model outputs are classified from a set of input patterns by learning from the past data and generalizing the lessons to predict future targets [128,129]. Case-based reasoning (CBR), Fuzzy Set Theory (FST), and Artificial Neural Networks (ANN) are common in AI to model the deterioration of infrastructure facilities [130,131].

CBR implemented deterioration prediction applications [118,132] are designed through a longitudinal and diversified database leading to the outputs of a query case. Future condition of a facility is modelled through the query case. Fuzzy logic has been developed to implement human logic, which comes through subjective categories [133]. Further extending the notion, FST has been employed to mathematically convert linguistic inference rules into fuzzy numbers and fuzzy rules [134]. Increased applications [135,136,137] in infrastructure deterioration modeling is due to the reduced subjectivity of trained data through fuzzy rules.

The principle of Neural Networks (NN) concerns the way the human brain performs with its densely interconnected set of nerve cells, called neurons [128]. ANN learns the patterns of the underlying process from the past data and generalize the gained knowledge to predict outputs [134]. ANN has also gained much attention in infrastructure deterioration assessment and management modeling [138,139,140]. Neuro Fuzzy System (NFS) has been defined by combining FST and ANN to collate the strengths and minimize the weaknesses of both methods [128,134]. NFS has widely been applied for the assessment and management of deterioration in different infrastructures [141,142].

Decision making plays a major role for the owners to manage their infrastructure assets in a sustainable way [113]. Decision making can be entirely based on financial criteria depending on the core aspect of their asset management plan based on financial assessment [113]. Accordingly, these organizations adopt an optimized decision making process based on benefit–cost analysis (BCA), which involves quantifying and comparing benefits and/or costs over a period of time using the net present value (NPV) method. Madanat outlines the basis of maintenance and rehabilitation decisions in four categories: available budget, the cost and effectiveness of different activities, the current and projected levels of usage, and the condition of assets [143].

An advanced asset management plan with a higher level of information about the assets enables decision-makers to base their decisions not only on financial aspects, but also on social, environmental, and cultural aspects. This decision-making process is called multi-criteria decision making (MCDM). Baker et al. 2001) identify the general decision-making process comprised of eight major steps: (1) define the problem, (2) determine requirements, (3) establish goals, (4) identify alternatives, (5) define criteria, (6) select a decision-making tool, (7) evaluate alternatives against criteria, (8) validate solutions against problem statement [144]. MCDMs have been employed in many decision making applications [145] while [146] used MCDM for sustainable management of community buildings in Australia.

**Figure 13:**
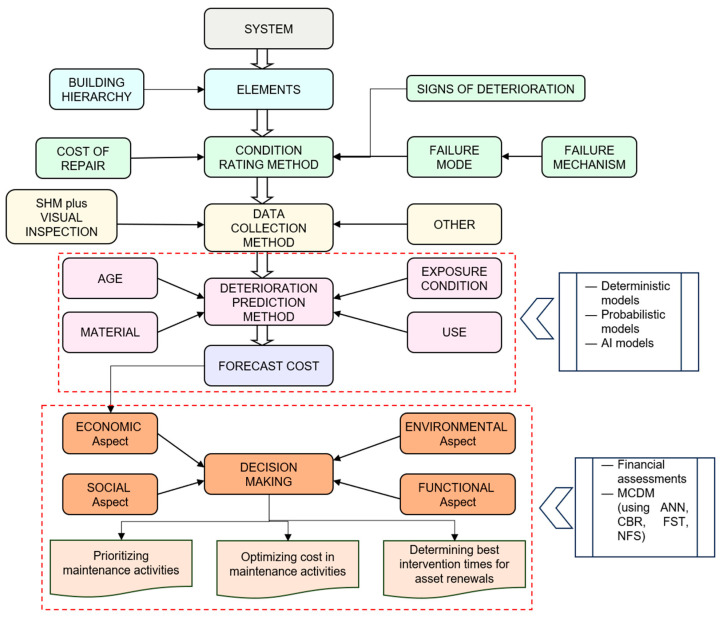
Suggested asset management framework.

In summary, asset management system comprises of deterioration prediction model followed by a decision making model. Deterioration prediction model requires an effective way to rate condition of elements. The common collection of condition data is relied on visual inspection, which is based only on appearance. Although different condition ratings are defined the condition inspection can be subjective. These condition data can always misinterpret the remaining useful life in the absence of actual structural health monitoring. SHM can be a reliable and objective way to collect data for existing asset management systems and these data can be trained as a mechanism for effective deterioration prediction. Considering the critically analyzed literature review data of this paper and aligned with the building management framework proposed by Kalutara [147], SHM embedded objective asset management framework is suggested in Figure 13.

Suggested framework starts with an asset management system which consists of elements. Identifying a whole system through elements is significant for informed asset management decision-making, because an asset element represents the level of asset management approach. Established element hierarchy should be chosen from the current practice depending on the asset type, whether a building or an infrastructure. Once the element hierarchy is finalized, condition rating method should be established to identify the condition of elements. An effective condition rating method should focus on signs of deterioration, cost of repair and the failure mode considering potential failure mechanisms. Destructive and non-destructive mechanisms can be used for the collection of data based on the selected condition rating method. Non-destructive mechanisms are preferred considering the damage and cost incurred by destructive mechanisms. Hence, visual inspection is widely used as non-destructive mechanisms. However, unreliable visual inspection data to measure structural integrity leads to engage reliable mechanisms such as application of structural health monitoring systems.

Combined mechanisms of visual inspection and SHM will pave the path for deterioration prediction of the given elements. As previously explained, trained data will be used to develop deterioration prediction models using deterministic, probabilistic or AI methods. Cost forecast is mainly targeted through the deterioration prediction model, which is connected with the last step of the framework—‘Decision-making’. Sustainable asset management decisions are planned to make through a decision making model which can be developed utilizing financial assessments and MCDM methods (ANN, CBR, FST and NFS). Sustainable decisions are based on triple bottom line aspects (environment, society, and economy) along with functional aspect. The developed model will enable making sustainable decisions:

- In prioritizing maintenance activities;
- Of optimizing cost in maintenance activities;
- In determining best intervention times for asset renewals.

## 6. Conclusions

The study was committed to identify non-destructive ways to perform SHM of structures; hence, a comprehensive review of sensor technologies was conducted for this purpose. This provided a collection of possible technologies including ultrasound sensors, mechanical sensors, laser sensors, optical sensors, infrared thermographic sensors, ground penetrating-radar sensors, electrical parameter measuring sensors and micro electromechanical systems sensors. The awareness of technologies prompted the study to identify the applications automated with these technologies; thus, another detailed review was conducted on automated systems for SHM. The study then discussed the theories, concepts and applications largely based on those automated systems such as AI, ML, and DT. SHM was commonly intended for assets; hence, the next focus of the study was the possible practical implementation of the previously reviewed findings in asset management. This enabled us to identify the proper utilization of SHM in the data collection process. Then, we discovered that the deterioration of assets can be predicted through a deterministic or probabilistic or AI model using those data. The outputs of deterioration prediction models can be used to forecast cost required for lifting the conditions of assets to better conditions within a given time frame. Asset managers can make decisions based on that economic aspect plus other sustainable aspects including environment, social, and functional aspects. Such decisions can be automated through the combination of financial assessments and MCDM applications. The paper has significantly contributed to the current asset management practice through the proposed asset management framework by relevantly collating all the reviewed findings.

## Figures and Tables

**Table 1:** Classification of concrete quality.

Pulse Velocity (km/s)	Quality of Concrete
>4.5	Excellent
3.5–4.5	Good
3.0–3.5	Medium
<3.0	Poor

Source: IS13311-1:1992.

**Table 2:** Recently developed electrical sensors for SHM.

Sensor Function	Materials/Construction	Measurement Mechanism	Output Parameter(s)	Applications	Ref
Impact Sensor	PZT/standard PZT sensor	LRC	Voltage (amplitude)	Aerospace SHM	[33]
Crack Detector	Dielectric material/Cylinder	Reflection Coefficient and spectrum shift	S_11_	Surface crack detection on metallic structures	[35]
Strain Sensor	Cu printed on Flexible substrate/Double layer flat coils on a magnetostrictive layer	Guided wave	Voltage (amplitude)	Defect detection in metallic planar structures	[42]
Crack Detector	PZT/standard PZT sensor	LRC	Voltage (amplitude, and phase)	Surface crack detection on metallic structures	[34]
Crack Detector	UHF Antenna	Reflection Coefficient and spectrum shift	S_11_	Surface crack detection on metallic structures	[36]
Strain Sensor	PDMS/IDT	Capacitance	Capacitance, Frequency	Strain measurement on surfaces	[39]
Strain Sensor	Standard strain gauge	Resistance	Voltage	Strain measurement on surfaces	[40]
Strain Sensor	PVDF/IDT metal deposited on PVDF	Capacitance	Capacitance	strain measurement on surfaces	[41]
Crack Detector	Taconic TLX-0/circular antenna and Meandered transmission line	Reflection Coefficient and spectrum shift, Time domain reflectometry	S_11_, Voltage amplitude	Surface crack detection on metallic structures	[37]
Crack Detector	PZT/standard PSI-5H4E sensor	Impedance	Magnitude, phase angle	Surface crack detection of structures	[44]
Strain Sensor	FR4/printed rectangular microstrip antenna	Reflection Coefficient and spectrum shift	S_11_	Strain measurement on surfaces	[38]

**Table 3:** MEMS applications in SHM.

MEMS Sensor Domain	Application
Acceleration/vibration/Sound/Strain/Stress/Load	➢ Crack formation and progression in bridges and concrete structures, which can happen due to varying rates and amounts of drying, shrinkage, loading, creep, and other thermophysical effects.
Temperature	➢ Monitoring temperature during setting and curing of concrete mix. ➢ Thermal cracking due to uneven thermal distribution. ➢ Hydration process during curing
Moisture	➢ Detecting the presence of water or vapors present in cracks which can cause accelerated corrosion and carbonation. ➢ Structural integrity due to volumetric changes during freeze–thaw cycles

## Data Availability

Not applicable.
